# Immune Thrombocytopenia in Previously Healthy Individuals Following SARS-CoV-2 Vaccination (COVID-19 Immunization): A Descriptive Research of 70 Instances With a Focus on Biomarkers, Predictive Outcomes, and Consequences

**DOI:** 10.7759/cureus.26480

**Published:** 2022-07-01

**Authors:** Kamal Sharma, Smeet Patel, Zeel Patel, Kalpen B Patel, Darshini B Shah, Jinish Doshi, Priyank Chokshi, Chandan Sharma, MohmadSabir M Amdani, Ansh Parabtani, Urva Benani, Ashwati Konat

**Affiliations:** 1 Cardiology, Dr. Kamal Sharma Cardiology Clinic, Ahmedabad, IND; 2 Medicine, Smt Nathiba Hargovandas Lakhmichand (NHL) Municipal Medical College, Ahmedabad, IND; 3 Medicine, Ahmedabad Municipal Corporation's Medical Education Trust (AMC MET) Medical College, Ahmedabad, IND; 4 Medicine, Pandit Deendayal Upadhyay Medical College, Rajkot, IND; 5 Medicine, Gujarat Cancer Society (GCS) Medical College, Hospital & Research Center, Ahmedabad, IND; 6 Internal Medicine, Ahmedabad Municipal Corporation's Medical Education Trust (AMC MET) Medical College, Ahmedabad, IND; 7 Internal Medicine, Pandit Deendayal Upadhyay Medical College, Rajkot, IND; 8 Medicine, Government Medical College, Surat, IND; 9 Internal Medicine, Sheth Lallubhai Gordhandas Municipal General Hospital, Ahmedabad, IND; 10 Internal Medicine, Gujarat Adani Institute of Medical Sciences, Bhuj, IND; 11 Internal Medicine, Smt Nathiba Hargovandas Lakhmichand (NHL) Municipal Medical College, Ahmedabad, IND; 12 Zoology, Biomedical Technology and Human Genetics, Gujarat University, Ahmedabad, IND

**Keywords:** thrombosis, vaccination, covid-19, sars-cov-2, immune thrombocytopenia (itp)

## Abstract

The coronavirus disease-2019 (COVID-19) pandemic is exacerbating the worldwide healthcare crisis. The pandemic has had an impact on nearly every system of our body. The Food and Drug Administration (FDA) gave immediate authorization of several vaccines to avoid critical COVID-19 outcomes following the rapid spread of the COVID-19. There have only been a few cases of severe acute respiratory syndrome coronavirus 2 (SARS-CoV-2) vaccination-induced immune thrombocytopenia (ITP) so far. There should be enough information to identify whether some vaccination adverse effects, such as ITP, are caused by the vaccine. This study aims to determine how common ITP occurs after receiving the SARS-CoV-2 vaccine, as well as gender, age, symptoms, biomarkers, predicted outcomes, and sequelae. We looked at a number of research and compiled the best evidence of SARS-CoV-2 vaccine-induced thrombocytopenia currently available. To find the recommended reporting items, the search technique included keywords like "Immune thrombocytopenia," "COVID-19," "SARS-CoV-2," and “Vaccination.” The search results were grouped using Boolean operators ("OR," "AND").

## Introduction and background

In December 2019, the first instance of coronavirus disease-2019 (COVID-19) was detected in Wuhan, China. It has a larger prevalence, a longer incubation time, and the ability to spread asymptomatically, on the other hand. As of April 8, 2022, COVID-19 had been documented in 494,587,638 cases over the world, with 6,170,283 deaths [1]. Worldwide, healthcare systems are accelerating vaccination efforts to protect the public from the severe acute respiratory syndrome coronavirus 2 (SARS-CoV-2) virus. Many modest and temporary adverse effects have been reported following the administration of several SARS-CoV-2 vaccines around the world. Thrombocytopenia is a probable side effect of SARS-CoV-2 vaccination, which may limit vaccine use due to a lack of pathophysiologic evidence. ITP is a type of acquired hemorrhagic disease marked by a platelet count of less than 100 × 10^9^/L as a result of immune-mediated platelet breakdown, reduced generation, or increased splenic sequestration [2]. The majority of instances of ITP are idiopathic, with just a few cases recorded as a result of many other triggering events such as immunizations, drugs, or infections. The Winton Centre for Risk and Evidence Communication at the University of Cambridge evaluated the benefits and side effects of the SARS-CoV-2 vaccine and discovered that the risk of clotting is roughly 1:250,000 in the general population, though it is 1.1:100,000 in young persons (20-29 years old) [3].

According to the German Society of Thrombosis and Haemostasis, 31 occurrences of sinusitis or cerebral vein thrombosis were reported to the Paul Ehrlich Institute out of about 2.2 million AstraZeneca COVID-19 vaccine doses distributed. The thromboses appeared 4-16 days following COVID-19 vaccine inoculation, and 19 people had thrombocytopenia, with nine people dying as a result of the clinical course. The patients ranged in age from 20 to 63 years, with two men aged 36-57 years [4]. In four instances, antibodies activated platelets via the Fc receptor, demonstrating a striking resemblance to heparin-induced thrombocytopenia [4].

In this current study, we looked at the current situation of SARS-CoV-2 vaccine-induced immune thrombocytopenia (ITP) and its possible pathogenesis, symptoms, biomarkers, repercussions, and recovery rate. The objective of this research is to learn as much as possible about the epidemiology and symptoms of COVID-19 vaccine-induced ITP.

## Review

Methods

We looked at case reports and case series that indicated ITP after COVID-19 immunization, independent of vaccine type, and we ruled out any other COVID-19 vaccine-related adverse events that had been published online. We discovered 20 studies that fulfilled our criteria, including 70 cases of ITP following COVID-19 vaccination [5-24] (Table 1).

**Table 1:** Case reports and case series that were used as references in this investigation. COVID-19, coronavirus disease 2019; SARS-CoV-2, severe acute respiratory syndrome coronavirus 2; ITP, immune thrombocytopenia; PE, pulmonary embolism; HIT, heparin-induced thrombocytopenia.

No	Authors	Brief Description
1	Preethi Suresh et al. [5]	According to the researchers, thrombocytopenia is caused by an immune response that is similar to a rare heparin-like immune response and has a link to PF4 antibodies. As a result, D-dimer, platelet, fibrinogen, and PF4 heparin antibody ELISA tests are necessary.
2	Andreas Tiede et al. [6]	Women aged 41-67 years who presented some days after their preliminary COVID-19 vaccination were included in this study. Different patients had symptoms such as cerebral venous sinus thrombosis, multiple cortical emboli and aortic arch thrombi, headache, and visual disturbance. Low platelet counts and elevated D-dimer levels plagued the patients.
3	Luigi Angelo Vairaa et al. [7]	The researchers give the example of a patient who had a small bluish lesion on his cheek removed. Blood testing revealed severe secondary ITP, which was most likely caused by the COVID-19 immunization, which was given to individuals three days prior to the onset of the disease.
4	Anne Louise et al. [8]	A 30-year-old woman developed thrombocytopenia and several thromboses after ingesting the COVID-19 vaccination, according to the authors. Autoimmune HIT was suspected due to a high 4T HIT score and a positive anti-PF4 antibody ELISA.
5	Syed Raza Ali Shah et al. [9]	Because there are few cases and statistics, it is critical to keep an eye on those who have had ITP and spare the second vaccination dosage for those who do not.
6	Marcello Candelli et al. [10]	A patient in this trial had thrombocytopenic purpura shortly after immunization against COVID-19, and vaccine-induced ITP was hypothesized. In authors' opinion, the rarity of these incidents has little influence on the overall benefits of immunization.
7	Sudhamshi Toom et al. [11]	A patient with significant thrombocytopenia after immunization against COVID-19 with mRNA vaccine was included in the study. Moreover, the fact that platelet counts improved despite continuing usage of a combined contraceptive refutes the hypothesis that it was the cause.
8	Katharina Guetl et al. [12]	According to this case report, a 50-year-old lady had vaccine-induced immunological thrombotic thrombocytopenia after receiving the main dose of COVID-19 immunization.
9	Kerry J. Welsh et al. [13]	According to the study, the rate of thrombocytopenia was 0.80 per million doses. There have been very few cases of vaccine-induced ITP recorded after SARS-CoV-2 vaccination.
10	Jay Hocking et al. [14]	According to this study, patients who develop thrombotic symptoms after receiving COVID-19 vaccine should be examined for thrombocytopenia, elevated D-dimer, and/or reduced fibrinogen, as well as anti-PF4 ELISA testing in the brain, central venous sinus, and abdomen.
11	Andreas Greinacher et al. [15]	Immune thrombotic thrombocytopenia caused by platelet-activating antibodies against PF4 may develop after vaccination with ChAdOx1 nCov-19. This clinically mimics autoimmune HIT.
12	Nina H. Schultz et al. [16]	After receiving primary vaccination against COVID-19, five patients had venous thrombosis and thrombocytopenia. Despite no prior heparin exposure, all of the patients had significant levels of antibodies against platelet factor 4–polyanion complexes.
13	Bilal Malik et al. [17]	Rare thromboembolic occurrences were documented in this case series, which were related with thrombocytopenia and occurred a few days after the COVID-19 immunization.
14	Juhaina Salim Al-Maqbali et al. [18]	A middle-aged woman suffered deep vein thrombosis and PE after receiving preliminary vaccination against COVID-19 illness with mRNA vaccine.
15	Fehmida Bano et al. [19]	The SARS-CoV-2 vaccination caused three cases of severe venous thrombosis, two of which resulted in fatal intracerebral hemorrhage and one of which resulted in PE. All three patients had high D-dimer values and low fibrinogen levels when they were admitted.
16	Luís Lourenço Graça et al. [20]	This study discovered an unexpected instance of widespread abdomen arterial and venous thrombosis shortly after receiving the COVID-19 vaccination, which has been linked to thrombosis
17	Leonor Dias et al. [21]	According to the study, two cases of cerebral venous thrombosis occurred shortly after receiving the mRNA vaccination. As a result, despite the fact that both cases occurred after vaccination, neither kind of cerebral venous thrombosis appears to be pathophysiologically distinct from other types of cerebral venous thrombosis.
18	Bader Al Rawahi et al. [22]	After having his first COVID-19 vaccination shot, a middle-aged man went to the emergency room with an intermittent fever and a dull nonspecific stomach ache.
19	Sareesh Bandapaati et al. [23]	A 50-year-old man developed celiac and splenic artery thrombosis after receiving the first dose of Oxford immunization, according to the study.
20	Azhar Kareem Razzaq et al. [24]	Patients with prior thrombocytopenia should be properly managed, according to this study, because the vaccine's immunological modulation process can aggravate pre-existing thrombocytopenia.

Search strategies

With the help of Google and scholarly resources like Google Scholar and PubMed, a comprehensive search of COVID-19 vaccine-related ITP case reports were carried out. Keywords like "Immune thrombocytopenia," "COVID-19," "SARS-CoV-2," and "Vaccination" are used in the search to locate items that should be reported. Boolean operators were used to group the search results ("OR," "AND").

Process of data collection and data items

The data was extracted individually by the authors using standardized data extraction forms. On an Excel sheet (Microsoft Corporation, Redmond, WA), we collected age, sex, and other variables such as type of vaccination, clinical manifestations, how long since they were detected with ITP, possible outcome, laboratory investigations, diagnosis strategies, and findings, and we reviewed all of these parameters.

Results

**Figure 1:**
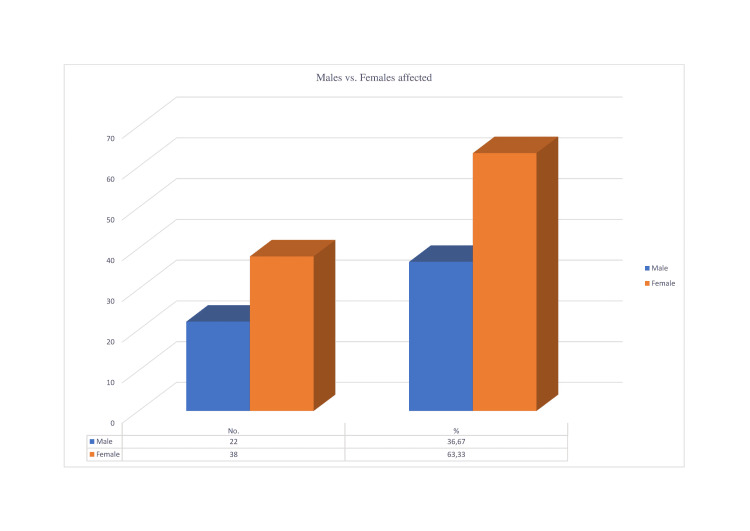
Percentage of males and females affected

The typical age of presentation was 50 years in this research of 70 published studies of COVID-19 vaccine-induced ITP (Interquartile range [IQR]: 38.25-61). Males account for 36.7% of reported cases, while females account for 38. (In 10 documented cases, the gender was not specified.) (Figure 1).

**Figure 2:**
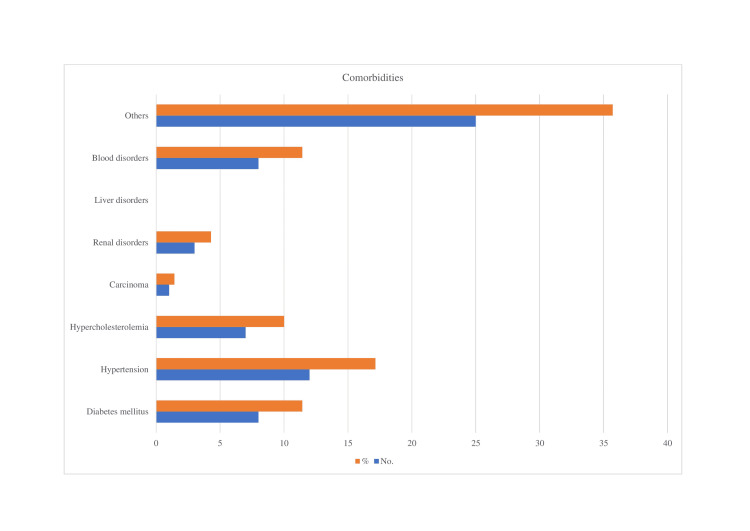
Types of comorbidities associated with the reported patients

In one family, there was a pre-existing blood disorder, a genetic disorder, and an autoimmune disease. None of the patients had indicated that they were addicted to any known substance of dependency. Details about any comorbidities that were present, such as diabetes, hypertension, hypercholesterolemia, cancer, renal diseases, liver problems, blood disorders, and others, are depicted in Figure 2.

**Figure 3:**
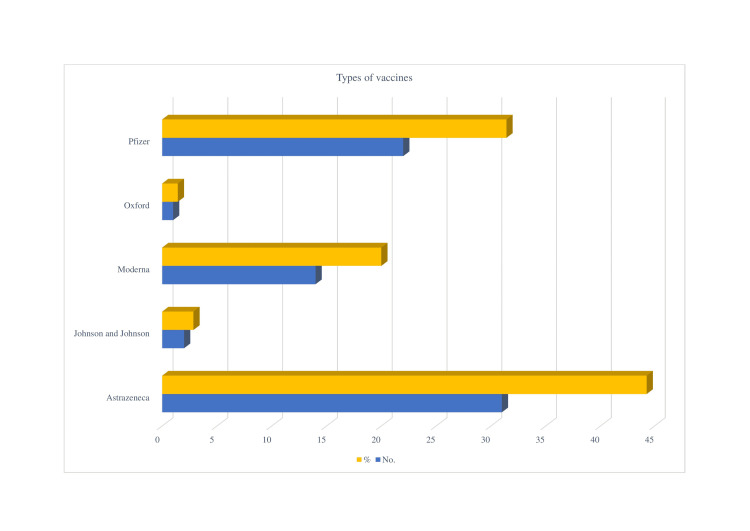
Percentage of immune thrombocytopenia with various types of vaccines

ITP incidences following vaccination with a variety of COVID-19 disease vaccines from Pfizer, Moderna, Oxford, AstraZeneca, and Johnson & Johnson (Figure 3).

**Figure 4:**
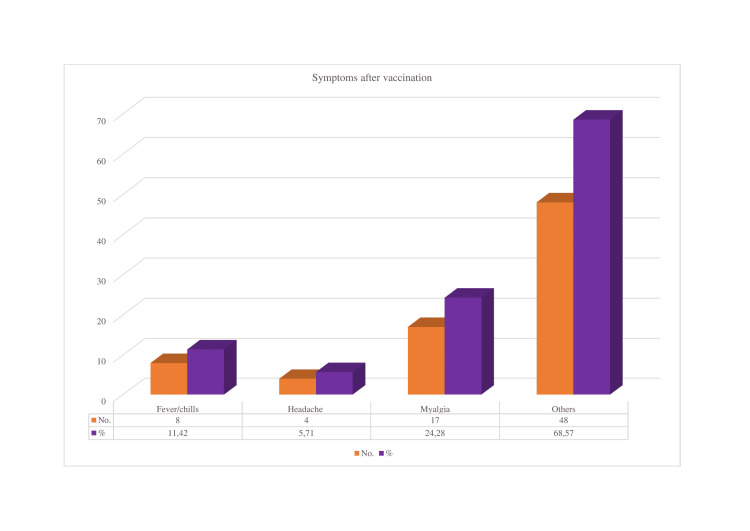
Symptoms developed after COVID vaccination

Fever, chills, headache, myalgia, and other symptoms were noted after immunization in our study (Figure 4).

**Figure 5:**
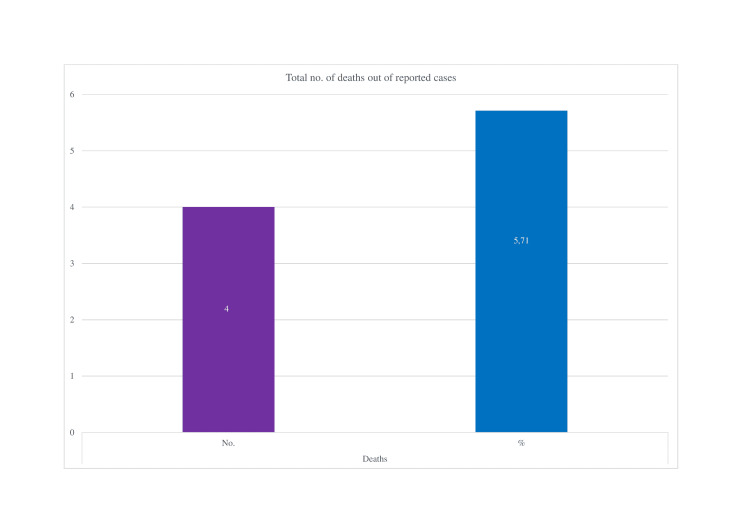
Total reported deaths after COVID vaccination

In our review, we found four deaths out of 70 reported instances (5.71% out of total reported cases) (Figure 5).

In our investigation, we discovered that the average time interval between the day of immunization against COVID-19 and the onset of illness was five days (IQR: 3-9 days).

**Figure 6:**
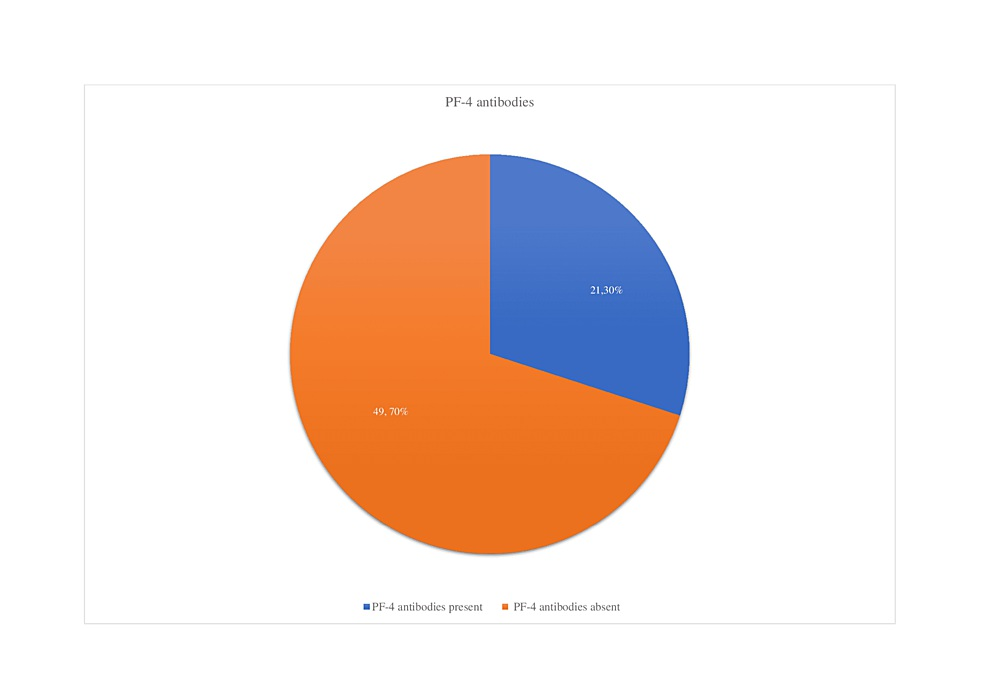
Findings of PF-4 antibodies in the reported cases

Out of 70 patients, we discovered that the PF-4 antibody was synthesized or present in 21 cases (30% of the total patients) (Figure 6).

**Figure 7:**
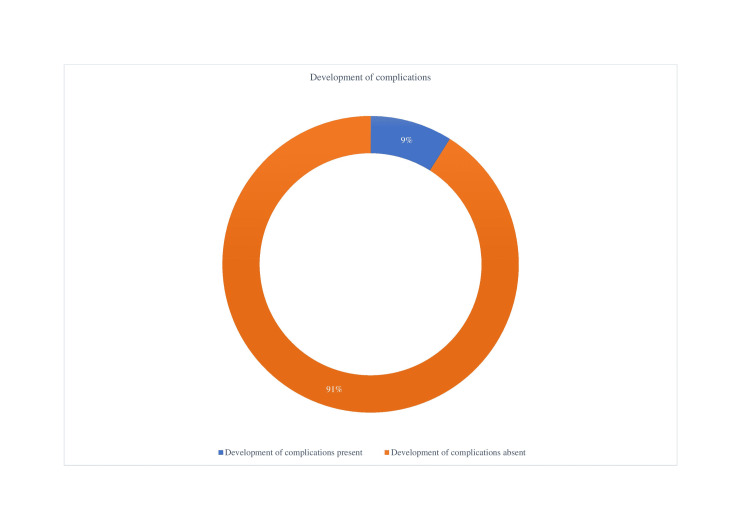
Total no. of patients who developed complications

In our analysis, we reported that six patients out of 70 had developed complications such as supraventricular tachyarrhythmia and arterial occlusion, accounting for approximately 9% of total cases (Figure 7).

Discussion

Since the World Health Organization (WHO) designated COVID-19 a pandemic on March 11, 2020, millions of individuals have been infected all across the world. Secure and dependable preventative vaccines were vital in halting the outbreak, which had major medical, economic, and cultural consequences. COVID-19 vaccine development and immediate usage approval is a big step forward toward disease prevention and minimizing morbidity and mortality.

ITP is a condition in which the immune response destroys platelets by mistake. It can be caused by HIV, hepatitis, or *Helicobacter pylori* infection in adults. ITP is a condition that affects children after a viral infection such as the mumps or the flu [25].

T-cells, a kind of WBCs, target platelets directly in a limited number of cases. Medication-induced allergies that cross-react with platelets could be the source of this immune system malfunction. Antibodies that cross-react with platelets can be caused by infections - most common viral diseases such as the viruses that cause chicken pox and hepatitis C, also with the immune diseases like rheumatoid arthritis and lupus, lymphomas, and leukemias, and even pregnancy [26].

ITP can be caused by both the MMR vaccine and the hepatitis B vaccine. The MMR vaccination (measles, mumps, and rubella) is a live vaccine that is given to children between the ages of 12 and 15 months and then again between the ages of four and six years. ITP after MMR vaccination is uncommon, with just one to four cases per 100,000 doses delivered. ITP, which emerges six weeks following receiving the MMR vaccine, is common [27].

The significant increase in proinflammatory cytokines such as interleukin (IL)-6, IL-8, and tumor necrosis factor-alpha (TNF-a) in severely ill SARS-CoV-2 patients implies that cytokine storm formation is crucial for COVID-19 clinical progression [28,29]. In the current circumstances, monoclonal antibodies against IL-6 receptors (such as tocilizumab) aid health care in COVID-19-associated pneumonia patients [30,31].

Researchers have yet to discover the exact relationship between SARS-CoV-2 vaccination and the incidence of thrombocytopenia. Only 17 cases of ITP were detected among the more than 20 million patients in the United States who got dose of the Pfizer and Moderna SARS-CoV-2 vaccinations as of February 2, 2021 [32,33]. According to the estimates, one de novo incidence would occur for every million vaccination recipients, which is consistent with the zero occurrences recorded in the joint Pfizer and Moderna vaccine trials, which included over 70,000 participants [32,33].

Secondary ITP after many types of vaccines produces an unsettling scenario. According to one study, approximately one instance of ITP occurs in approximately 40,000 children who receive the MMR vaccine [34].

Because of a lack of precise pre-vaccination platelet counts and imprecise temporal case reporting, it is currently difficult to assess the true incidence rate of ITP following SARS-CoV-2 immunization. Despite the fact that existing data suggest that the frequency of ITP following SARS-CoV-2 immunization is roughly equivalent to contemporaneous cases after vaccination, this material should be viewed as preliminary.

## Conclusions

The present evidence shows that, while it is uncommon, young people without comorbidities are at an increased risk of developing ITP within 3-10 days of receiving the SARS-CoV-2 vaccine (COVID-19 vaccination). Early symptoms in the majority of cases include headaches, vision problems, lethargy, vomiting, shortness of breath, and petechiae. The majority of cases exhibited elevated D-dimer levels, while just a few had PF-4 antibodies. The majority of people experienced mild symptoms, with only a few developing complications, and the fatality rate was extremely low when compared to other idiopathic causes of ITP.
